# Purification, crystallization and preliminary X-ray crystallographic studies of Rv3705c from *Mycobacterium tuberculosis*

**DOI:** 10.1107/S2053230X14014113

**Published:** 2014-07-23

**Authors:** Feifei Lu, Feng Gao, Honglin Li, Weimin Gong, Lin Zhou, Lijun Bi

**Affiliations:** a Shanghai Key Laboratory of New Drug Design, School of Pharmacy, East China University of Science and Technology, 130 Meilong Road, Shanghai 200237, People’s Republic of China; b National Laboratory of Biomacromolecules and Laboratory of Noncoding RNA, Institute of Biophysics, Chinese Academy of Sciences, Beijing 100101, People’s Republic of China; c Center for Tuberculosis Control of Guangdong Province, Guangzhou, People’s Republic of China

**Keywords:** *Mycobacterium tuberculosis*, Rv3705c

## Abstract

The conserved protein Rv3705c from* Mycobacterium tuberculosis* has been cloned, expressed, purified and crystallized by the sitting-drop vapour-diffusion method using PEG 3350 as a precipitant. The Rv3705c crystals exhibited space group *P*6_1_22 or *P*6_5_22, with unit-cell parameters *a* = *b* = 198.0, *c* = 364.1 Å, α = β = 90, γ = 120°, and diffracted to a resolution of 3.3 Å.

## Abstract 2

The cloning, expression, purification, crystallization and preliminary X-ray diffraction analysis of Rv3705c from *M. tuberculosis* are described.

## 1. Introduction   

*Mycobacterium tuberculosis* (Mtb), the causative agent of tuberculosis, is estimated to have infected one-third of the world’s population (World Health Organization, 2013 ▶). Multi-drug-resistant (MDR) and extensively drug-resistant (XDR) Mtb strains are becoming more and more widespread, presenting a serious threat to tuberculosis control (Raviglione & Smith, 2007 ▶; Kliiman & Altraja, 2009 ▶). Accordingly, further research on Mtb is extremely urgent and important.

The complete genome of the best characterized strain of Mtb, H37Rv, has been sequenced and many of the open reading frames for its proteins have been annotated (Cole *et al.*, 1998 ▶; Camus *et al.*, 2002 ▶). Rv3705c is an Mtb protein with a molecular weight of 22.4 kDa comprised of 214 amino acids, the function of which is unknown. Rv3705c was identified to be a culture filtrate protein by two-dimensional PAGE combined with liquid chromatography-coupled MS/MS (Målen *et al.*, 2007 ▶; de Souza *et al.*, 2011 ▶). It is predicted to have an N-terminal 25-residue signal peptide by *SignalP* (Petersen *et al.*, 2011 ▶). A sequence-similarity search using commonly used programs such as *BLAST* (Altschul *et al.*, 1997 ▶) indicates that it has a PknH-like extracellular sensor domain, which is also found in lipoproteins LpqQ, LprH, LppH and LpqA from *M. tuberculosis*. The PknH sensor domain adopts a distinctive fold containing two intramolecular disulfide bonds and a large V-shaped cleft, suggesting that this domain binds a small-molecule ligand that signals by modulating the localization or oligomerization of the kinase (Cavazos *et al.*, 2012 ▶). In this paper, we report the cloning, expression, purification, crystallization and preliminary X-ray studies of Rv3705c.

## 2. Materials and methods   

### 2.1. Molecular cloning   

The gene encoding Rv3705c (UniProt accession No. I6XI06) was amplified by polymerase chain reaction with *M. tuberculosis* H37Rv genomic DNA as the template (Brosch *et al.*, 1998 ▶). Based on the experimental conditions tested, the presence of 10% dimethyl sulfoxide (DMSO) proved to be essential for PCR amplification. DMSO is a co-solvent that improves the denaturation of GC-rich DNA, consistent with the 65.6% GC content of *M. tuberculosis* genomic DNA (Cole *et al.*, 1998 ▶). The forward (5′-CGC**GGATCC**ATGCGAATCGCCGCCGCG-3′) and reverse (5′-CCC**AAGCTT**GCCCAGTGTGTTCTGCATTGCC-3′) synthetic oligonucleotide primers contained *Bam*HI and *Hin*dIII restriction sites, respectively. The PCR product was cloned into TT-pET-28a expression vector, which was modified from pET-28a (Novagen) and replaces the thrombin cleavage site with a *Tobacco etch virus* (TEV) protease cleavage site, thus adding an N-terminal MGSSHHHHHHSSGLEVLFQGPHMASMTGGQQMGRGS tag and a C-terminal KLAAALEHHHHHH tag. The recombinant plasmid was transformed into *Escherichia coli* BL21(DE3) competent cells for expression. Positive clones were confirmed by DNA sequencing.

### 2.2. Protein expression and purification   

*E. coli* BL21(DE3) recombinant cells were grown at 310 K in LB medium to an OD_600_ of 0.6 and IPTG was then added to the culture medium to a final concentration of 0.4 m*M* to induce protein expression at 289 K. After 16 h, the cells were harvested by centrifugation at 4000*g* for 15 min at 277 K. The cells were resuspended in lysis buffer (20 m*M* Tris–HCl pH 7.9, 500 m*M* NaCl, 5 m*M* imidazole) and lysed by sonication. All subsequent steps were performed on ice or at 277 K. The cell lysate was centrifuged at 15 000*g* for 40 min at 277 K. The supernatant was applied onto an Ni–NTA affinity column containing 7 ml Ni Sepharose (GE Healthcare) pre-equilibrated with lysis buffer for 1 h. Ni Sepharose captures the target His-tagged fusion protein from the supernatant. The bound protein was eluted from the column with 30 ml elution buffer consisting of 20 m*M* Tris–HCl pH 7.9, 500 m*M* NaCl, 500 m*M* imidazole. Subsequently, the eluted protein was concentrated and buffer-exchanged to a buffer consisting of 20 m*M* Tris pH 7.9, 100 m*M* NaCl using a Millipore Amicon concentrator with a 10 kDa cutoff membrane. The protein sample was loaded onto a Q-Sepharose Fast Flow (2.6 × 8.2 cm) anion-exchange column (GE Healthcare) and fractionated using a linear gradient of 0.1–1 *M* NaCl. Fractions containing the target protein were pooled, concentrated and further purified to homogeneity by gel-filtration column chromatography using a Superdex 200 pg 16/60 column (GE Healthcare) with a buffer consisting of 20 m*M* Tris–HCl pH 7.9, 100 m*M* NaCl.

### 2.3. Crystallization   

Prior to crystallization, the protein sample was concentrated to 10 mg ml^−1^ using a Millipore Amicon concentrator with 10 kDa cutoff membrane. The protein concentration was determined by the method of Bradford (1976 ▶). Initial crystallization trials were carried out at 289 K using the sitting-drop vapour-diffusion method with screens from Hampton Research and Rigaku (Crystal Screen, Crystal Screen 2, PEG/Ion, PEG/Ion 2, Index, Wizard I and Wizard II). Screening for crystallization conditions was achieved using sitting-drop vapour diffusion in 96-well Intelli-Plates (Hampton Research) by mixing 200 nl protein solution with 200 nl reservoir solution and equilibrating against 35 µl reservoir solution. Crystals appeared in several conditions. Further manual screening in 24-well plates (1+1 µl drops, 200 µl reservoir solution) was performed to optimize the best condition based on the diffraction status of the crystals: 0.2 *M* lithium sulfate monohydrate, 0.1 *M* Tris pH 8.5, 25% PEG 3350 (Index condition No. 77). Refinement of the crystallization conditions was achieved by altering the precipitant concentration and the pH. The best diffracting crystals were grown with 0.2 *M* lithium sulfate monohydrate, 0.1 *M* Tris pH 8.8, 15% PEG 3350.

### 2.4. Data collection and processing   

For X-ray data collection, after successive soaking in cryoprotectant solutions consisting of mother liquor with increasing PEG 3350 content (final concentration of 20%) and 30% glycerol, crystals were flash-cooled in liquid nitrogen. Diffraction data were collected on beamline BL17U at the Shanghai Synchrotron Radiation Facility (SSRF), People’s Republic of China. A total of 180 frames were collected with an oscillation step of 1°and 0.6 s exposure per frame. The crystal-to-detector distance was maintained at 400 mm. Diffraction data were processed and scaled using *HKL*-2000 (Otwinowski & Minor, 1997 ▶). A summary of the X-ray diffraction data and processing statistics is given in Table 1 ▶.

## 3. Results and discussion   

**Figure 1:**
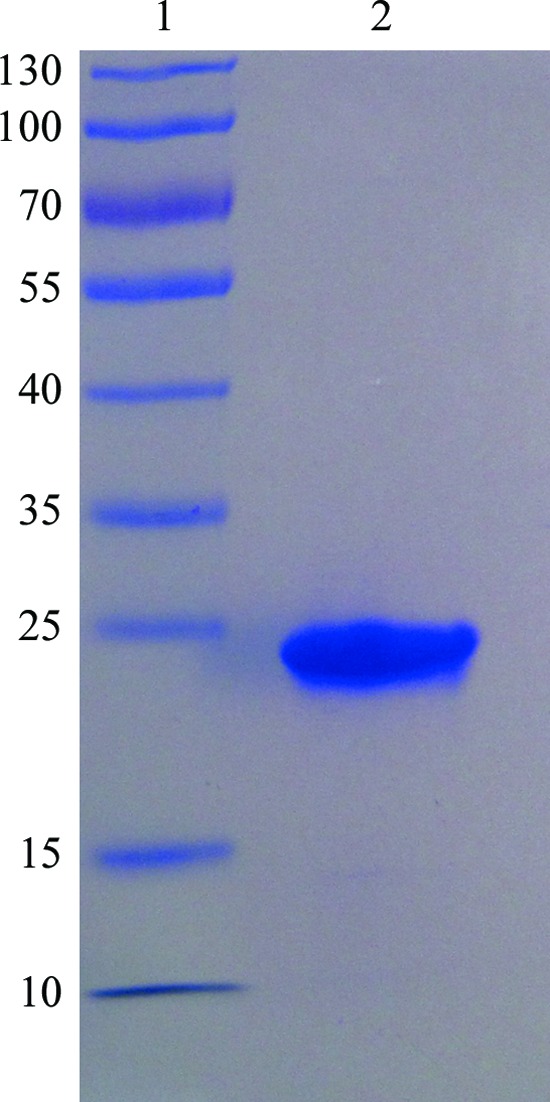
SDS–PAGE of purified Rv3705c. Lane 1, molecular-weight markers (labelled in kDa); lane 2, Rv3705c after gel-filtration column chromatography.

Rv3705c was successfully overexpressed in *E. coli* BL21(DE3) cells and a crystallization-grade sample was obtained by three purification steps consisting of nickel-affinity chromatography, anion-exchange chromatography and size-exclusion chromatography (Fig. 1 ▶). The yield of the pure protein was ∼10 mg per litre of culture.

**Figure 2:**
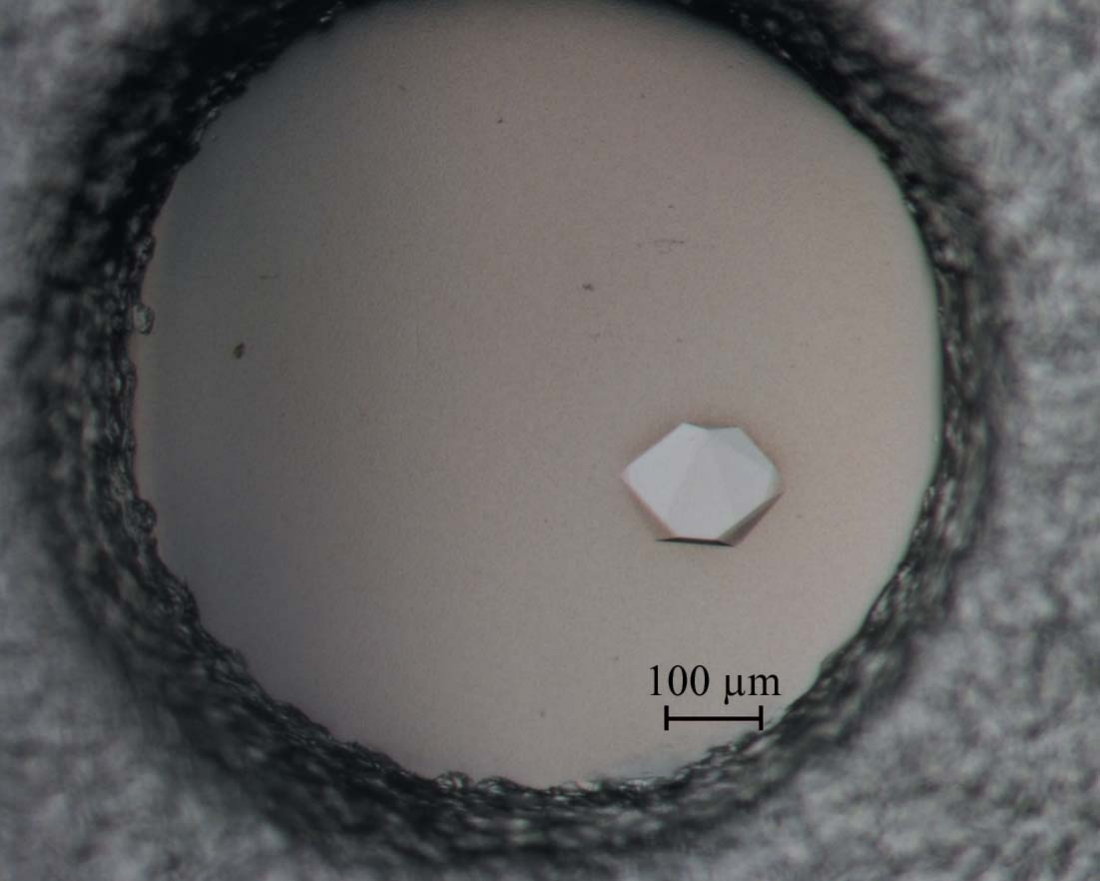
Crystal of Rv3705c.

**Figure 3:**
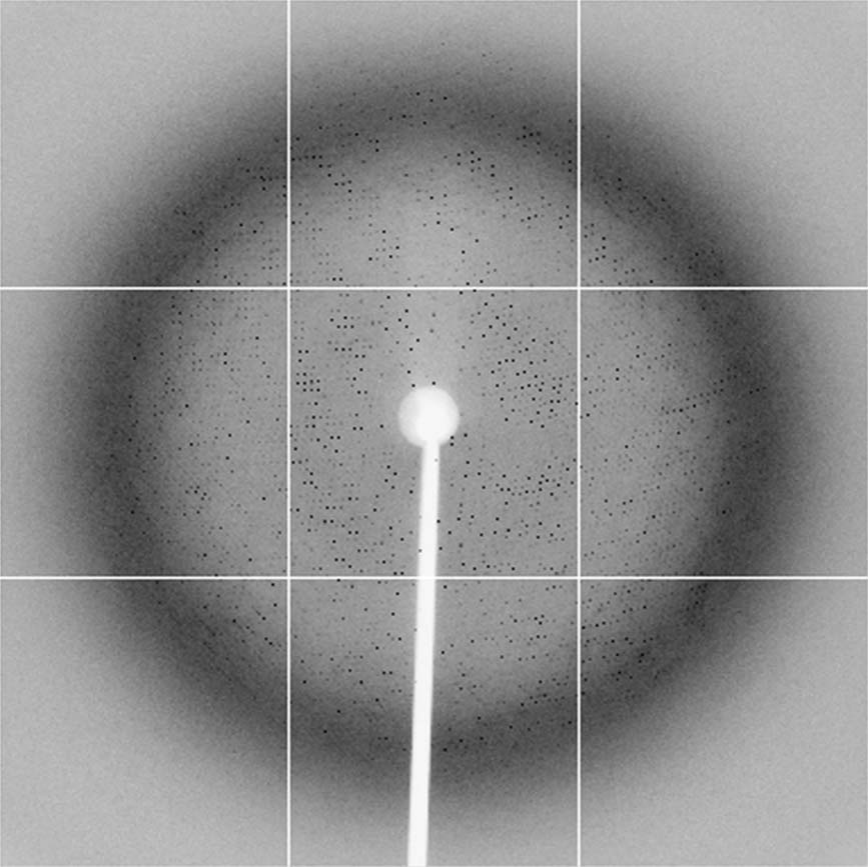
X-ray diffraction pattern of Rv3705c.

Initial crystals of Rv3705c appeared after about 5 d under several conditions. Various optimization procedures such as varying the precipitant concentration and the buffer pH were used in order to obtain diffraction-quality crystals. Finally, crystals were obtained using the sitting-drop vapour-diffusion method with reservoir solution consisting of 0.2 *M* lithium sulfate monohydrate, 0.1 *M* Tris pH 8.8, 15% PEG 3350 (Fig. 2 ▶). We chose Rv3705c crystals for N-terminal sequencing. The results showed that Rv3705c began with HPSEP, indicating that the crystallized protein was Rv3705c^26–214^, which was consistent with *E. coli* processing of the signal peptide. The crystals diffracted to 3.3 Å resolution (Fig. 3 ▶) and belonged to space group *P*6_1_22 or *P*6_5_22. The unit-cell parameters of the native crystal were *a* = *b* = 198.0, *c* = 364.1 Å, α = β = 90, γ = 120°. According to the unit-cell parameters and the molecular weight of Rv3705c, there could be up to 20 protein molecules in the asymmetric unit. The elution volume of the gel-filtration experiment showed that Rv3705c may form heptamers or octamers. Self-rotation function calculation showed a strong peak at (ϕ = 63.5°, ψ = 0°, κ = 104.2°). Weaker peaks for κ values of 155.2° and 52.5° were also found. These peaks indicated a sevenfold symmetry. If there were a heptamer in the asymmetric unit, the corresponding *V*_M_ value should be 5.67 Å^3^ Da^−1^ (Matthews, 1968 ▶). Although no obvious twofold symmetry other than the crystallographic symmetry was found, two heptamers in the asymmetric unit appeared more reasonable.

Molecular replacement using *Phaser* (McCoy *et al.*, 2005 ▶) with the structure of PknH (PDB entry 4esq; Cavazos *et al.*, 2012 ▶) from *M. tuberculosis* as a search model (28% sequence identity for residues 66–161) was unsuccessful. In order to determine the structure of Rv3705c by single-wavelength anomalous diffraction (SAD) methods, a selenomethionyl derivative of Rv3705c is being prepared.

## Figures and Tables

**Table 1:** Summary of data-collection statistics for Rv3705c Values in parentheses are for the highest resolution shell.

Space group	*P*6_1_22 or *P*6_5_22
Unit-cell parameters (Å, °)	*a* = *b* = 198.0, *c* = 364.1, α = β = 90, γ = 120
Resolution range (Å)	50–3.3 (3.42–3.30)
Total No. of reflections	1390879
No. of unique reflections	64048
Completeness (%)	99.8 (100)
Multiplicity	21.7 (22.2)
Mean *I*/σ(*I*)	24.1 (8.4)
*R*_merge_ † (%)	14.2 (54.1)

† *R*_merge_ = 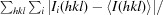 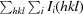, where *I_i_*(*hkl*) is the *i*th observation of the intensity of the unique reflection *hkl* and 〈*I*(*hkl*)〉 is the average over symmetry-related observations of the unique reflection *hkl*.
